# Liquid biopsy in gastrointestinal stromal tumors: a novel approach

**DOI:** 10.1186/1479-5876-12-210

**Published:** 2014-08-14

**Authors:** Margherita Nannini, Annalisa Astolfi, Milena Urbini, Guido Biasco, Maria A Pantaleo

**Affiliations:** Department of Specialized, Experimental and Diagnostic Medicine, Sant’Orsola-Malpighi Hospital, University of Bologna, Bologna, 40138 Italy; “Giorgio Prodi” Cancer Research Center, University of Bologna, Bologna, 40138 Italy

**Keywords:** Gastrointestinal stromal tumor, Mutational analysis, KIT, Platelet-derived growth factor receptor alpha, Circulating tumor DNA, Liquid biopsy

## Abstract

The role of molecular analysis in the management of gastrointestinal stromal tumors (GIST) remains indisputable. To date, tumor tissue extracted from specimens obtained by surgical or biopsy procedures has been the only source of the tumor DNA required for the molecular and genomic assessment of cancer. However, tumor tissue sampling has several clinical limitations: for example, the invasiveness of these procedures precludes repeated sampling. Thus, it is possible to obtain only a static molecular picture of the disease, a picture that lacks the inter- and intra-metastatic molecular heterogeneity that characterizes most GIST. In contrast, circulating tumor DNA obtained from a patient’s bloodstream, known as liquid biopsy, can theoretically overcome the limitations of tissue biopsies and provide the same molecular and genomic information. GIST are recognized as a paradigm of molecular biology among solid tumors. Although few but promising data on liquid biopsy in GIST have been accumulated to date, these tumors may provide the optimal field for application of this challenging approach.

## Introduction

The role of molecular analysis in the management of gastrointestinal stromal tumors (GIST) remains unexplored [1, 2]. Knowledge of mutational status provides clinicians with a mandatory guide to therapeutic decision making concerning GIST patients with advanced disease [3–5]. Mutational analysis has also been advocated in the adjuvant setting [6–8]: a recent trial on the relationship between tumor genotype and benefits of adjuvant imatinib reported that GIST with a *KIT* exon 11-deletion respond positively to treatment, with a significantly longer recurrence-free survival compared with placebo. However, this benefit was not observed for GIST *KIT* exon 11 point mutations and insertions, or *KIT* exon 9 or *KIT/platelet-derived growth factor receptor alpha (PDGFRA)* wild type (WT) [2].

To date, tumor tissue extracted from specimens obtained by surgical or biopsy procedures has been the only source of the tumor DNA required for the molecular and genomic assessment of cancer. However, such tumor tissue sampling has several clinical limitations: because it is obtained by invasive procedures that can have complications, it cannot be repeated frequently. In the absence of serial samples for analysis, only a static molecular picture of the disease, a picture that lacks the inter- and intra-metastatic molecular heterogeneity characteristic of most GISTs, is available [9, 10]. Moreover, tumor tissue in formalin-fixed paraffin-embedded blocks is not optimal for wide genome analysis, which requires high-throughput technologies for which fresh DNA is more suitable.

In the scenario under review here, circulating tumor DNA (ctDNA) obtained from a patient’s bloodstream, known as *liquid biopsy*, can theoretically overcome all the limitations of tissue biopsies and provide the same molecular and genomic information [11]. Because blood sampling is minimally invasive, it can be performed at any time during the course of the disease, allowing dynamic assessment of molecular changes in tumors over time. Moreover, it can provide the fresh tumor DNA needed for whole genome and exome analyses.

ctDNA investigations in cancer patients are increasingly being performed, supporting the different potential applications of this approach. Preliminary studies in patients with melanoma, ovarian, breast, prostate, and colon cancers have shown that the amount of ctDNA correlates with the tumor burden, suggesting a potential role for ctDNA as a surrogate marker of tumor response to treatment [12–16]. Additionally, detection of ctDNA after radical surgery or curative therapies indicates the presence of minimal residual disease and may thus identify patients who will experience tumor recurrence [12]. Other studies have shown that serial analysis of ctDNA during treatment can provide a dynamic picture of molecular disease changes, suggesting that this noninvasive approach could also be used to monitor the development of secondary resistance and identify heterogeneous subclonal populations of tumor cells developing during the course of treatment [17]. Such a monitoring application should be extremely useful during treatment of cancer with tyrosine-kinase inhibitors (TKIs). Finally, different strategies for detecting novel chromosomal alterations in plasma, such as rearrangements and amplification or epigenetic changes, have recently been developed, suggesting that ctDNA could also be used for detecting new tumor-derived variants for genotyping purposes [18–20].

## Review

Because the application of liquid biopsy in GIST has only been reported in the last year, few but promising data are now available [21, 22]. The feasibility of liquid biopsy and its application in GISTs was reported for the first time at the 2013 ASCO Annual Meeting [21, 22]. Using both Sanger sequencing on tumor tissue and Beads Emulsion Amplification Magnetics technology on plasma, Demetri *et al*. have analyzed the kinase genotypes in a subgroup of GIST patients in a phase III GRID trial [21]. They found 84% concordance between plasma and tissue for detection of primary *KIT* mutations. Additionally, they more easily detected secondary mutations in plasma (47%) than in tissue (12%). In contrast, the assay was less sensitive for the detection of primary *KIT* exon 11 mutations in plasma DNA (12% vs 43%), this apparent discrepancy being partly attributable to the study design, which was specifically targeted to secondary mutations, but mostly to the extensive heterogeneity of primary *KIT* exon 11 mutations that hampers the development of specific assays for each possible mutation carried by the tumor. These findings highlight a possible limitation of liquid biopsy that is shared by all other technologies specifically designed to identify *KIT/PDGFRA* mutations in GIST management; namely, the wide variability of tyrosine kinase mutations. Thus, less targeted approaches may be better able to provide a complete sequence of the hotspot exons such as CAPP-Seq or TAm-Seq. However, even in this scenario the very large insertions and deletions barely detected by massively parallel sequencing would constitute a drawback [23, 24]. Maier *et al*. retrospectively used 25 different allele-specific L-polymerase chain reaction assays covering *KIT* and *PDGFRA* mutations to examine 291 plasma samples from 38 GIST patients and correlated the detection of mutated ctDNA with disease status (active disease vs complete response [CR]) [22]. Interestingly, they found that, in the clinical setting, more patients with active disease, defined as patients having at least one lesion progressing or responding to treatment, had positive results than did CR patients without evidence of residual disease after surgery. They also examined whether the amount of ctDNA correlated with tumor radiological response and reported repeated positive test results and increasing mutant ctDNA in patients with disease progression, negative to positive conversions in patients with relapse, and positive to negative conversions in patients responding to TKIs. Taken together, these findings indicate that mutant ctDNA can be detected and quantified in the plasma of GIST patients and, notably, that the amount of mutant ctDNA correlates with tumor response, suggesting that this approach is feasible and can be used as a surrogate biomarker for predicting both tumor response and relapse in GIST patients.

Although very few data have been accumulated to date, liquid biopsy seems to be a promising tool in GIST management, offering a wide spectrum of clinical applications. The well-known kinase genotype and its relationship with tumor response to TKIs has made GIST a paradigm of molecular biology among solid tumors and provides a strong rationale for applying liquid biopsy in this disease. In particular, the possibility of taking serial blood samples to assess real-time molecular modifications during the course of treatment may identify the development of heterogeneous resistant clones, thereby optimizing the timing of changes in therapeutic strategy. However, because the therapeutic armamentarium available for advanced GIST is limited, this noninvasive approach should not be applied only to anticipate changes in treatment, but also to guide clinicians in re-challenging with drugs according to a rotation strategy.

In addition to offering a dynamic picture of molecular changes during the course of a disease that could serve as early biomarkers of tumor response to TKIs, other intriguing applications of liquid biopsy in GIST management are possible. First, it could be used to assess minimal residual disease after radical resection of a primary tumor, thus accurately identifying patients at high risk of recurrence for whom adjuvant treatment is indicated. Plainly, the main limitation of this application is the ability of the methods used to detect very small amounts of ctDNA; more sensitive assays should be developed for this purpose. Moreover, in the light of the extreme biological heterogeneity of GIST, especially those without the known *KIT/PDGFRA* or *BRAF/RAS/NF1* or *SDH* mutations (termed WT GIST), liquid biopsy may be the best tool for obtaining a wide molecular picture of GIST by using whole genome analyses with high-throughput technologies, such as next-generation sequencing approaches. ctDNA could be used as a source for identifying novel chromosomal adaptations such as rearrangements and translocations, thus overcoming the limitations of creating fresh tissue banks. This would facilitate a more detailed molecular classification of this disease and shed light on new diagnostic and prognostic biomarkers and eventually new potential targets, thereby extending the therapeutic armamentarium for a subset of GIST. Finally, liquid biopsy could also be used to select patients for clinical trials according to their molecular profile, rather than relying solely on the standard clinical and pathological features adopted to date.

## Conclusions

Liquid biopsy is emerging as one of the most challenging and promising tools in oncology. Because GIST are recognized as the paradigm of molecular biology in solid tumors, these tumors represent an optimal field in which to use this approach.

## Abbreviations

PDGFRA: Platelet-derived growth factor receptor alpha
GIST: Gastrointestinal stromal tumor
ctDNA: Circulating tumor DNA
WT: Wild type
TKI: Tyrosine-kinase inhibitor.
